# Temperature-Dependent Electrical Transport Properties of Individual NiCo_2_O_4_ Nanowire

**DOI:** 10.1186/s11671-018-2844-3

**Published:** 2019-01-08

**Authors:** Caihong Jia, Feng Yang, Lei Zhao, Gang Cheng, Guanghong Yang

**Affiliations:** 1 Henan Key Laboratory of Photovoltaic Materials, School of Physics and Electronics, Henan University, Kaifeng, 475004 People’s Republic of China; 2 Key Lab for Special Functional Materials of Ministry of Education, Henan University, Kaifeng, 475004 People’s Republic of China

**Keywords:** NiCo_2_O_4_ nanowires, Electrical transport properties, Schottky emission, Variable range hopping model, Nearest neighbor hopping model

## Abstract

Understanding the electrical transport properties of individual nanostructures is of great importance to the construction of high-performance nanodevices. NiCo_2_O_4_ nanowires have been investigated widely as the electrodes in electrocatalysis, supercapacitors, and lithium batteries. However, the exact electrical transport mechanism of an individual NiCo_2_O_4_ nanowire is still ambiguous, which is an obstacle for improving the performance improvement of energy storage devices. In this work, NiCo_2_O_4_ nanowires were prepared successfully by thermal transformation from the CoNi-hydroxide precursors. The electrical transport properties of an individual NiCo_2_O_4_ nanowire and its temperature-dependent conduction mechanisms were studied in detail. The current-voltage characteristics showed that an ohmic conduction in a low electrical field (< 1024 V/cm), Schottky emission in a middle electric field (1024 V/cm < *E* < 3025 V/cm), and Poole–Frenkel conduction at a high electric field (> 3025 V/cm). A semiconductive characteristic is found in the temperature-dependent conductivity in the NiCo_2_O_4_ nanowire; the electrical conduction mechanism at low temperature (*T* < 100 K) can be explained by Mott’s variable range hopping (VRH) model. When the temperature is greater than 100 K, electrical transport properties were determined by the VRH and nearest neighbor hopping (NNH) Model. These understandings will be helpful to the design and performance improvement of energy-storage devices based on the NiCo_2_O_4_ nanowires.

## Introduction

High-performance energy storage devices are the key to the development of new energy vehicles, large-scale energy storage, and micro-/nano-devices [1, 2]. The current energy-storage devices, including lithium batteries and supercapacitors mainly based on carbon electrodes devices, have many limitations such as a low efficiency at the first cycle, no discharge plateau, poor cycling performance, and the serious voltage delay in charge-discharge curves [3–5]. Generally, the structures and properties of the electrodes in energy storage device directly determined the performances of energy storage devices [6]. Therefore, it is crucial to find and design a new electrode that possesses superior power density, high capacity, and good cyclability for practical applications.

Nickel-cobalt oxides are one of multifunctional transition metal oxide semiconductor materials [7, 8]. Recently, it aroused great research interests as a promising candidate electrode material for energy storage devices due to its several inherent advantages such as low cost, environmental friendliness, high theoretical capacity [9, 10], good electrochemical activity, and better conductivity than nickel oxides or cobalt oxides [11, 12]. However, in practical applications, these energy storage devices based on metal oxide electrodes showed poor cycling performance due to these electrodes that cannot retain their integrity after few discharge-charge cycles. Nanostructured low dimensional materials often showed excellent physical properties due to their unique nanostructures, so engineering NiCo_2_O_4_ electrode at the nanoscale might help improve the electrode properties, such as enlarging the active surface areas, shortening the ion transport pathways, and relieving the strain status. Different nanostructured NiCo_2_O_4_ materials [13–15], especially nanowires/rods [16, 17] and their nanocomposites with carbon fibers, graphene, and porous Ni [18–27], have been studied extensively and the performances of energy storage devices have been improved such as ultrahigh specific capacitance, excellent cycling performance at high rates and excellent structural stability, etc. The electrical transport properties of nanostructured materials are crucial and determine their success or failure of applications for high-performance nanodevices. Nevertheless, NiCo_2_O_4_ nanowires/nanorods, as a basic building block most widely used in the fields of electrocatalysis, supercapacitors, and lithium batteries [16–27], their exact electrical transport mechanism is still ambiguous. To our knowledge, there are no reports concerned about the electrical transport properties of an individual NiCo_2_O_4_ nanowire. More importantly, the temperature has a significant impact on the ionic diffusion and electrical transport properties of the electrodes and the performance of energy storage devices [28]. So, the study of temperature-dependent electrical properties is helpful for clarifying the electrical transport mechanism in semiconductor electrode materials [29]. In this work, NiCo_2_O_4_ nanowires were synthesized successfully by thermal transformation from the CoNi-hydroxide precursors and the electrical transport properties and temperature-dependent conduction mechanisms of the individual NiCo_2_O_4_ nanowire device were studied systematically. With increasing the applied electrical field, the current-voltage characteristics can be explained by Ohmic mechanism, Schottky emission mechanism, and Poole–Frenkel conduction mechanism, respectively. The conduction process has been understood by employing conventional models, namely variable range hopping (VRH, *T* < 100 K) model and nearest neighbor hopping (NNH, *T* > 100 K) model. These understandings will be helpful to the design of energy-storage devices based on the NiCo_2_O_4_ nanowires.

## Methods/Experimental

### Synthesis of NiCo_2_O_4_ Nanowires

In a typical process [20], the CoNi ions-containing precursor solutions were prepared by dissolving the 1.19 g CoCl_2_·6H_2_O, 0.595 g NiCl_2_·6H_2_O, 0.728 g hexadecyl trimethylammonium and 0.54 g Co(NH_2_)_2_ into 50 mL DI water and this mixed solution was prepared under magnetic stirring for 30 min in air, and then the prepared solution was transferred to a Teflon-lined stainless steel autoclave. A piece of carbon cloth was firstly washed by ultra-sonication in ethanol and distilled water for 5 min, then dried in an oven, and finally immersed into an autoclave containing a 50 mL precursor solution. And the autoclave was kept at 100 °C for 12 h. After a hydrothermal process, the precipitation and carbon cloth with precursors was taken out and subjected to heat treatment at 300–380 °C in a muffle furnace for 3 h.

### Fabrication of Individual NiCo_2_O_4_ Nanowire Device

The Cr/Au electrodes were fabricated by a standard electron beam lithography (EBL) process. First, a certain amount of NiCo_2_O_4_ nanowires was put into ethanol and ultrasound for 3 min and then dispensed on a clean silicon wafer with a 200-nm-thick SiO_2_ layer. Second, a layer of 250-nm-thick polymethylmethacrylate (PMMA) was spin-coated on a silicon wafer and baked at 180 °C for 5 min. Next, focused electron beam on JSM 5600 scanning electron microscope was controlled to write the electrode patterns in PMMA films corresponding to the location of a NiCo_2_O_4_ nanowire. And then the exposed PMMA samples were developed imaging in the mixed solvent of methylisobutylketone and isopropanol (1:3) and fixed in isopropanol. Fourth, the developed sample was brought into the chamber of electron beam evaporation and resistance evaporation composite coating system (TEMD 500). When the vacuum level reaches 10^−4^ Pa, the Cr source was heated by electron beam and evaporated, the 5–10 nm Cr layer was deposited on the sample. And then, the Au source was heated by resistance wire and evaporated onto the sample, the thickness of Au film was about 70 nm monitored by an in situ film thickness detection system. Finally, PMMA layers were lifted off in acetone, only leaving two Au electrode pads at the ends of an individual nanowire.

### Characterization

The topographical images of NiCo_2_O_4_ nanowire samples were characterized by using a scanning electron microscope (SEM, Nova Nano SEM 450), a transmission electron microscope (TEM, JEM 2010), and atomic force microscope (AFM mode, Dimension Icon). UV-Vis absorption spectra were recorded using a (PE Lambda 950) spectrophotometer. The current-voltage (I-V) characteristics were recorded at room and low temperatures (CCR-VF, Lakeshore) system and a semiconductor parameter analyzer (Keithley 4200 Instruments, Inc).

## Results and Discussion

### Characterizations of NiCo_2_O_4_ Nanowire

**Fig. 1:**
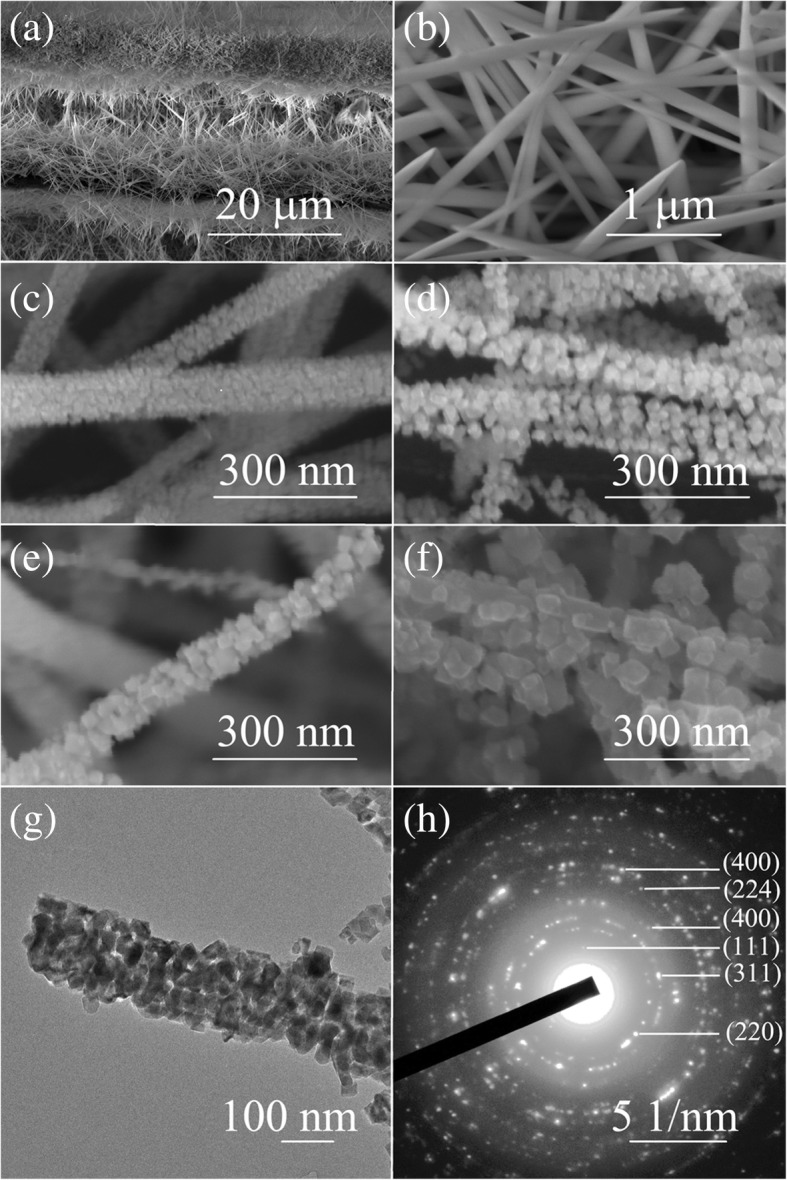
**a**, **b** The SEM image and enlarged one of precursor NiCo_2_(OH)_6_ nanowires. **c**–**f** The high-resolution SEM images of NiCo_2_O_4_ nanowires at annealing temperatures of 300 °C, 330 °C, 360 °C, and 380 °C. **g**, **h** The TEM image and selected-area electron diffraction pattern

The preparation method of NiCo_2_O_4_ nanowires is referred to the reported works [20], and the annealing at 300–380 °C converts the NiCo-precursor into spinel NiCo_2_O_4_ grown on the textiles by a simple oxidation reaction [20]. Figure 1a, b showed the SEM images of precursor NiCo_2_(OH)_6_ nanowire exhibiting smooth topography. Figure 1c–f presents higher-magnification SEM images of NiCo_2_O_4_ nanowires annealed at 300 °C, 330 °C, 360 °C, and 380 °C, respectively. It can be seen from the SEM images that when annealing at 300 °C, the nanowire surface became rough and crystallized into many small projections in diameter of about 20 nm. With increasing the annealing temperature, the grain size of the projections becomes increased and became about 90 nm at 380 °C annealing, as shown in Fig. 1f. The TEM image in Fig. 1g showed that annealed NiCo_2_O_4_ nanowires be composed of small crystalline grains; this kind of mesoporous structure is propitious to the penetration of electrolyte onto the surface of nanowires achieving rapid charge transfer reactions due to the short ion diffusion paths. The selected-area electron diffraction pattern [20] shows well-defined polycrystalline diffraction rings, which correspond to the (440), (224), (311), (111), (220), and (400) planes, as shown in Fig. 1h.

**Fig. 2:**
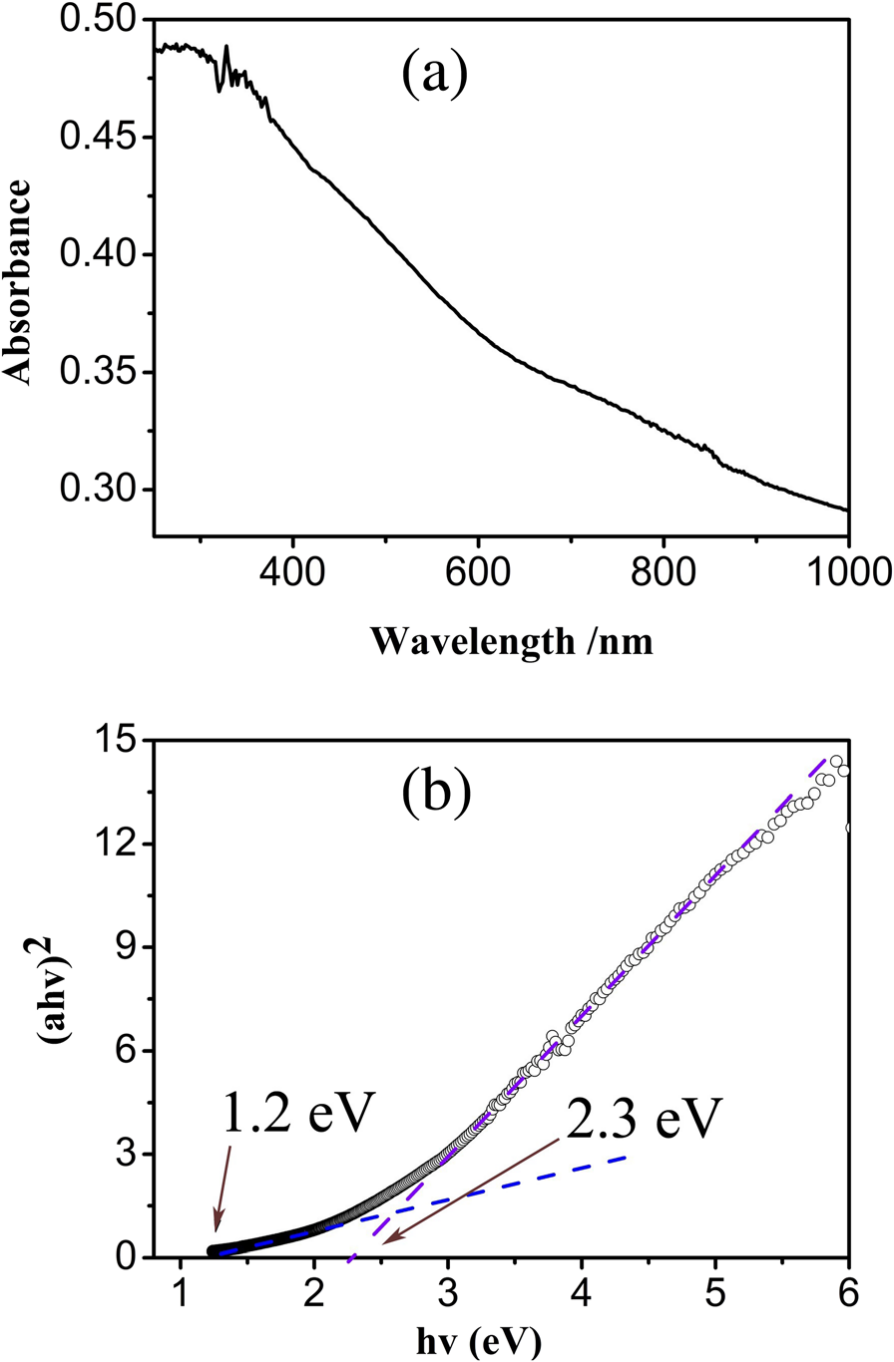
**a** UV-Vis absorption spectrum of the NiCo_2_O_4_ nanowires. **b** Optical band gap energy of NiCo_2_O_4_ nanowires obtained by extrapolation to (αhv)^2^ = 0

Figure 2a presents the UV-Vis absorption spectrum of the NiCo_2_O_4_ nanowires annealed at 300 °C. According to the relationship equation between the optical band gap and absorption coefficient of semiconductor materials, (αhv)^n^ = *K*(hv-E_g_), optical energy band gap (E_g_) can be derived. Here, hv is the photon energy, α is the absorption coefficient, *K* is a constant concerned about the materials, and *n* is related to the material and the electron transition types, here, the best fit gives *n* = 2 for the indirect bandgap semiconductor material. Figure 2b showed two absorption band gap energies, 1.1 eV and 2.3 eV, obtained by extrapolating the straight line segment to (αhv)^*n*^ = 0. The phenomenon of two absorption band gap has been studied and explained by the co-existence of high-spin and low-spin states of Co^3+^ in the NiCo_2_O_4_ nanowires [30]. Thus, the tetrahedral high spin Co^2+^, octahedral low spin Co^3+^, and Ni^3+^ exist in the electron configuration of NiCo_2_O_4_ nanowires. The band structure is defined by taking the O 2p orbital as the valence band and the Ni 3d, Co 3d orbitals as the conduction band. Including the electron transition from the O 2p orbital to high-spin Co 3d orbital, there exists the transition from low-spin orbital to the high-spin orbital of Co 3d owing to the partially filled band of high-spin states in NiCo_2_O_4_ nanowires. Therefore, the two band gaps were observed in the optical absorption spectrum. The value of the optical band gap is dependent on the sizes, micro-/nano-morphologies and structures, and crystal boundary of nanomaterials [31]. Table 1 presents a comparison of reported band gap values of NiCo_2_O_4_ nanostructures.

**Table 1:** Comparison of reported band gap values of different NiCo_2_O_4_ nanostructures

Nanostructures of NiCo_2_O_4_	The values of Eg (electron transition types)		Size dimension: *D* = diameter, *L* = length, *T* = thickness	
	O-2p orbital to high-spin Co-3d orbital	Low-spin to high-spin orbital of Co-3d		
Core-rings [30]	3.63 eV	2.06 eV	*D*: 80–150 nm	*T*: 10–20 nm
Nanoplates [32]	2.90 eV	1.80 eV	*D*: 3 μm	*T*: 70 nm
Nanowires	2.30 eV	1.20 eV	*D*: 200 nm	*L*: 3–5 μm

### The Electrical Transport Properties of Individual NiCo_2_O_4_ Nanowire

**Fig. 3:**
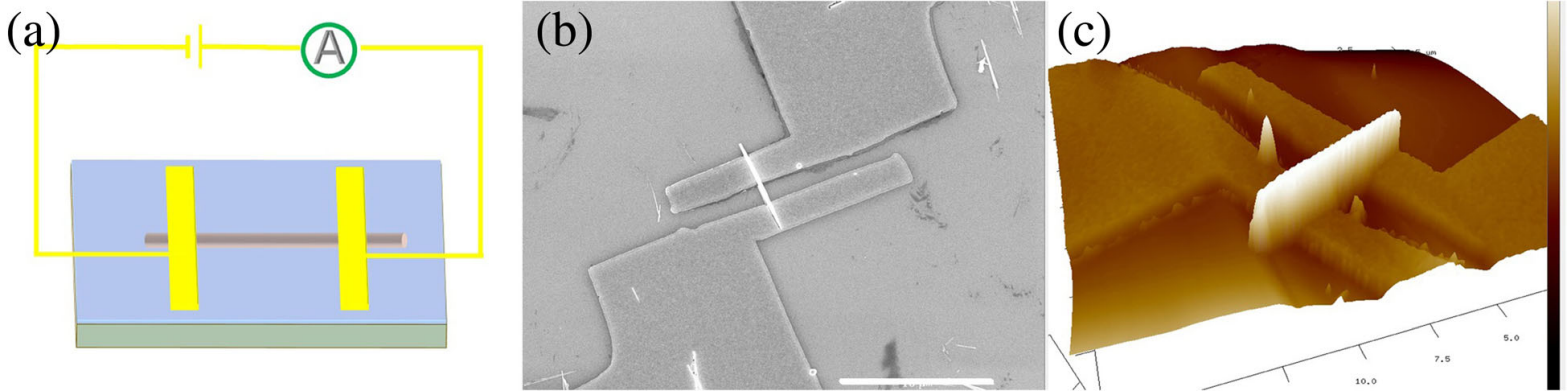
**a** The schematic illustration of individual NiCo2O4 nanowire device. **b**, **c** The SEM image and 3D AFM topographic image of the Au/Cr electrode pads

**Fig. 4:**
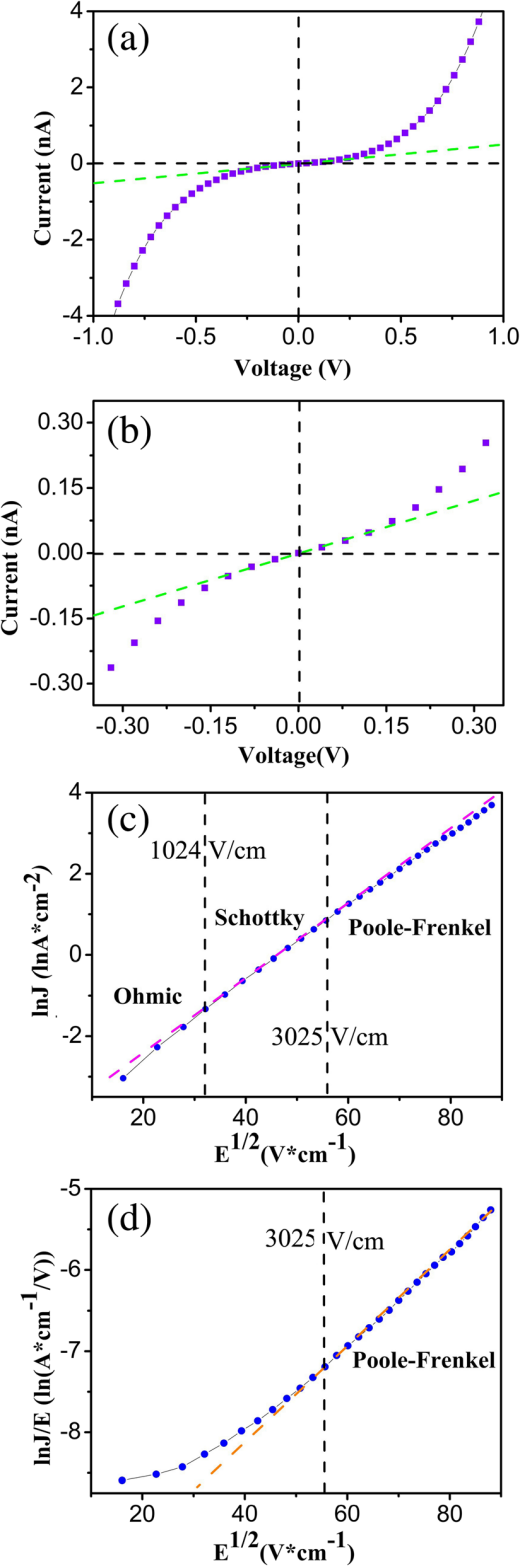
**a** The I-V curve individual NiCo_2_O_4_ nanowire device. **b** The enlarged image at low voltage values. **c** The plot of ln(J) vs E^1/2^ according to the Eq. (1). **d** The plot of ln(J) vs E^1/2^ according to the Eq. (3)

The electrical transport properties of nanostructured materials are crucial to their applications in high-performance nanodevices. Particularly, predictable controllable conductance is very helpful to design the nanoscale electrical components with precise regulation and control function. Therefore, we investigated the direct current conductivity and electrical transport mechanism of an individual NiCo_2_O_4_ nanowire. Figure 3a is the schematic illustration of individual NiCo_2_O_4_ nanowire device. Figure 3b, c gives the SEM image and 3D AFM topographic image of the Au/Cr electrodes on an individual NiCo_2_O_4_ nanowire, respectively. The I-V curve was performed at room temperature to investigate the electrical transport properties of an individual NiCo_2_O_4_ nanowire. As shown in Fig. 4a, b, the I-V curve characteristic is symmetrical and changes linearly for the applied voltages less than 0.15 V, which can be explained by the ohmic mechanism in the low electrical field.

Here, we consider the nanowire as a cylinder to obtain the cross-sectional area (*A*), *A* = $\uppi \ast {\left(\frac{D}{2}\right)}^2$. The conductivity value (σ) can be obtained by the formula $\upsigma =\frac{I}{U}\ast \frac{L}{A}$, where *L* and *A* denotes the length and cross-sectional area of the NiCo_2_O_4_ nanowire, respectively. According to Fig. 3b, c, the effective length (*L*) of NiCo_2_O_4_ nanowire, the distance between the two electrodes, is about 1.55 μm, and the nanowire diameter (*D*) is about 188 nm from the AFM image. Therefore, the conductivity of the nanowire, σ ≈ 0.48 S cm^−1^, can be derived by assuming that the contact resistance is zero. This value is close to the conductivity of polycrystalline NiCo_2_O_4_ (σ ≈ 0.6 S cm^−1^) reported in Fujishiro’s works [8], but Hu et al. [32] reported the higher conductivity (σ ≈ 62 S cm^−1^) of single-crystal NiCo_2_O_4_ nanoplates. In Fujishiro’s work, polycrystalline NiCo_2_O_4_ was prepared from the powder precursor materials at an annealing temperature of 900–1000 °C, and the large grain was composed of numerous small grains with many grain boundaries, so the electron transport will be affected by grain boundary scattering. The electron transport in a single crystal was free of grain boundary scattering effect and a larger conductivity of the nanowires were prepared from NiCo-hydroxides precursor by the annealing treatment at 300 °C, and their SEM and TEM images showed that the NiCo_2_O_4_ nanowire is a porous nanowire composed of many small nanoparticles of 10–20 nm in diameter instead of the single-crystal nanowire, similar to the case in Fujishiro’s work. Therefore, a conductivity value, close to that of polycrystalline NiCo_2_O_4_, was obtained in our works.

As shown in Fig. 4a, the current increases exponentially with the applied voltage increasing when the voltage is larger than 0.15 V. The current versus voltage in semiconductor nanostructures are discussed in several conduction mechanisms [33, 34] including Schottky emission, Poole–Frenkel (P-F) emission, Fowler–Nordheim tunneling, and a space charge limited current. In order to determine the dominant electrical transport mechanism, the logarithm of the current density is plotted against the square root of the electric field, as shown in Fig. 4c; a straight line at the electric field ranges from 1024 to 3025 V/cm suggests the Schottky emission. The Schottky current density is expressed as follows[32–34]:

1 $$ \ln J=\frac{\beta_{SE}}{kT}\sqrt{E}+\left[\ln A{T}^2-\frac{q\varnothing }{kT}\right] $$

Here, *A* is a constant, ∅ is the Schottky barrier height, *q* is the electron charge, *k* is Boltzmann’s constant, and *E* is the electric field. The constant *β*_*SE*_ is given as follows:

2 $$ {\beta}_{SE}=\sqrt{\frac{q^3}{4\pi {\varepsilon}_0{\varepsilon}_r}} $$

Here, *ε*_0_ is the permittivity of the free space and *ε*_*r*_ is the relative dielectric constant. The relative dielectric constant value (*ε*_*r*_ ≈ 18.7) obtained according to the slope is larger than that reported value (*ε*_*r*_ ≈ 11.9) of single-crystalline NiCo_2_O_4_ nanoplates [32], which may be due to the polycrystalline characteristics in our individual NiCo_2_O_4_ nanowires.

With the increase of the electrical field (*E* > 3025 V/cm), the J-E curve characteristic agrees well with the P-F transport mechanism, as shown in Fig. 4d. The Schottky transport mechanism is explained by thermo-electron emission of the free charge carriers, but the P-F transport denotes emission from structural defects in active traps, which is expressed by the following formula [33, 34]:

3 $$ \ln \frac{J}{E}=\frac{\beta_{PF}}{\mu kT}\sqrt{E}+\left[\ln C-\frac{q\varnothing }{\mu kT}\right] $$

Here, *q*∅ is the ionization potential in eV, denoting the amount of energy required for the trapped electron to overcome the influence of the trapping center when no field is applied. ${\beta}_{PF}\sqrt{E}$ is the amount by which the trap barrier height is reduced by the applied electric field E. *C* is a proportionality constant and *k* is the Boltzmann constant. The parameter *μ* is introduced in Eq. 3 for taking into account the influence of the trapping or acceptor centers (1 < *μ* < 2). For *μ* = 1, the conduction mechanism is considered as the normal P-F effect, whereas it is termed as the P-F effect with compensation or the modified P-F effect when *μ* = 2. In this case, the semiconductor contains a non-negligible number of carrier traps. The P-F constant is given by

4 $$ {\beta}_{PF}=\sqrt{\frac{q^3}{4\pi {\varepsilon}_0{\varepsilon}_r}} $$

Here, *ε*_0_ is the permittivity of the free space and *ε*_*r*_ is the relative dielectric constant. The relative dielectric constant *ε*_*r*_ ≈ 55.3 is extracted from the slope of the straight line region of the log(J/E) vs E^1/2^ curve according to the P-F emission.

Based on the above analysis, electrical transport can be explained by the ohmic mechanism of conductivity in the low electrical field (< 30 V/cm), with the increase of the applied electrical field (1024 V/cm < *E* < 3025 V/cm), the dominant conduction mechanism is determined to be Schottky emission. At the high electrical field (> 3025 V/cm), the dominant conduction mechanism fits well with the P–F conduction mechanism.

**Fig. 5:**
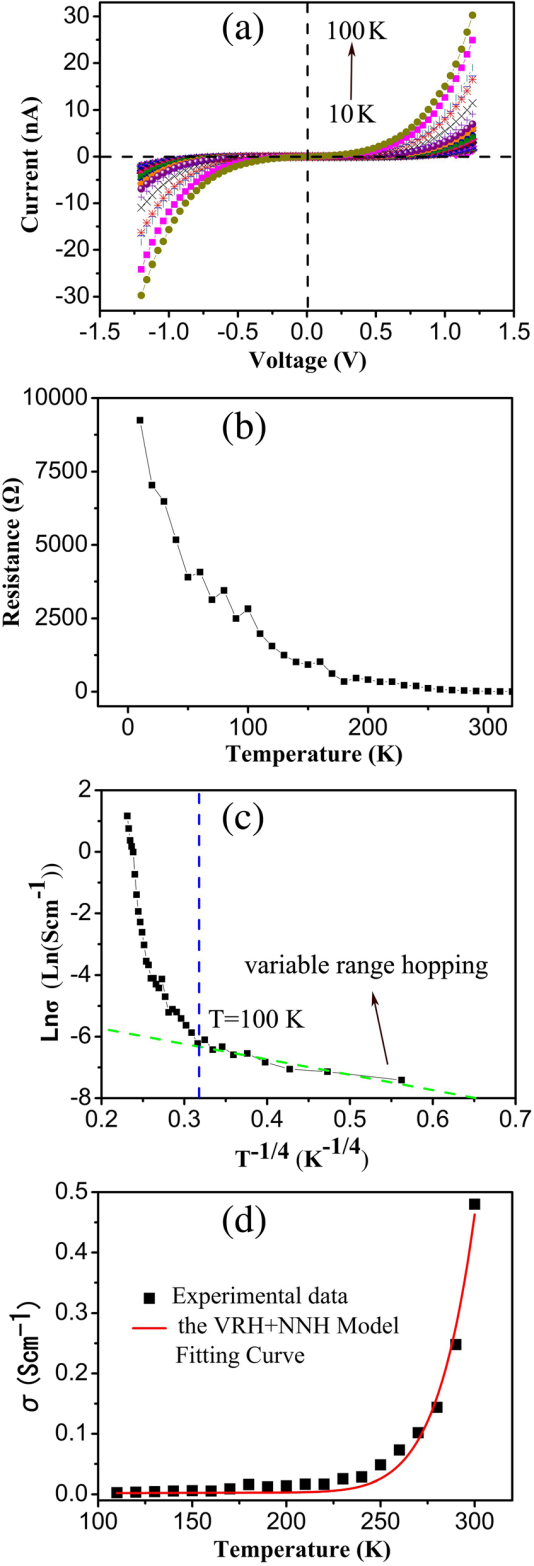
**a** The I-V curves with a temperature of 10 K to 300 K at intervals of 10 K. **b** The resistance versus temperatures. **c** A plot of lnσ as a function of *T*^-1/4^ and a fit to the NRH model when *T* < 100 K. **d** The plot of conductivity σ as a function of *T* when *T* > 100 K

The conductivity is dependent on the carrier concentrations and mobilities, both of which rely on the temperature. Therefore, the deeper study on the temperature dependence of conductivity is very important for understanding the electrical transport mechanism. In this work, temperature-dependent I-V characteristics were obtained in the range of 10–300 K at intervals of 10 K. As shown in Fig. 5a, b, the current values of both the forward and reverse biases increased rapidly with the temperature increasing, and the resistance (*R*) decreased exponentially with temperature (*T*) implying a typical semiconductive characteristic [35]. However, the change of conductivity σ with temperature does not accord with the thermal excitation mechanism defined by $\upsigma ={\sigma}_0\exp \left(-\frac{\Delta  \mathrm{E}}{\mathrm{kT}}\right)$, where *σ*_0_ is a constant and ∆E is the activation energy. As for the temperature-dependent conductivity, two typical hopping mechanisms, called variable range hopping (VRH), which happens at low temperatures, and nearest neighbor hopping (NNH), which takes place at high temperatures, have been proposed by Mott et al. for some semiconductor materials. The relationship between σ and *T* for the VRH and NNH mechanisms can be described by the following formula [35, 36]:

5 $$ {\sigma}_1={\sigma}_0\mathit{\exp}\left[-{\left(\frac{T_0}{\mathrm{T}}\right)}^{\frac{1}{4}}\right]\ \left(\mathrm{VRH}\right) $$

6 $$ {\sigma}_2=\left[\frac{\nu_0{e}^2c\left(1-c\right)}{\upkappa \mathrm{Tr}}\right]\exp \left(-2\upalpha \mathrm{r}\right)\exp \left(-\frac{\varDelta E}{kT}\right)\ \left(\mathrm{NNH},T>\mathrm{Debye}\ \mathrm{temperature}\right) $$

Here, *T*_*0*_ is the VRH temperature constant concerned on the density of localized states at Fermi energy, σ_0_ is a constant, *ν*_0_ is the longitudinal optical phonon frequency, α is the rate of wave-function decay, *r* is the average hopping distance, *c* is the fraction of sites occupied by electrons or polarons, and ΔE is the action energy. In our works, when the temperature was less than 100 K, the σ versus *T* accords well with the VRH model: *σ*_1_=0.016exp[$-{\left(\frac{1840}{T}\right)}^{\frac{1}{4}}$], here *σ*_0_ = 0.016, *T*_0_ = 1840, as shown in Fig. 5c. When the temperature was higher than 100 K, the σ-*T* relations according to the VRH and NNH models:

7 $$ \sigma ={\sigma}_1+{\sigma}_2=0.016\exp \left[-{\left(\frac{1840}{T}\right)}^{\frac{1}{4}}\right]+\frac{32086}{T}\exp \left[-\frac{0.0235}{\mathrm{k}T}\right] $$

The activation energy (*ΔE*) of the NiCo_2_O_4_ nanowire was calculated to be 0.0235 eV, less than the value reported for NiCo_2_O_4_ bulk (0.03 eV) [37] and single-crystal nanoplates (0.066 eV) [32].

According to our analysis, the VRH model dominates electrical transport at low temperatures. With the temperature increasing, both the VRH and NNH mechanisms play roles at a critical temperature of 100 K (Debye temperature). The hopping conduction mechanism implied the existence of surface or bulk defects, and vacancies in our NiCo_2_O_4_ due to its polycrystalline characteristics. In Mott’s mechanism, the conductivity of a semiconductor is resulting from the hopping of carriers in material, which is assisted by lattice vibrations (phonons) [36]. In the VRH hopping process, a hopping step may span a greater distance than that between nearest-neighbor-hopping sites, and the optical phonons do not have enough energy to assist the hopping at low temperature. So, the conduction mechanism in NiCo_2_O_4_ nanowire at low temperatures is an acoustic single-phonon-assisted hopping process according to Schnakenberg’s theory [38]. In the NNH model, optical-phonon-assisted hopping of small Polaris between localized sites is used to interpret the conduction mechanism. In NiCo_2_O_4_ nanowires, some small polaron can be considered as the holes or electrons localized at the lattice sites, and these localized carriers polarize their surrounding lattice, as a result, the coherent motion of free carriers through the lattice is disturbed and the carrier must hop between localized states [39].

## Conclusions

In this work, NiCo_2_O_4_ nanowires were prepared successfully by thermal transformation from the CoNi-hydroxide precursors and the electrical transport mechanisms of the individual NiCo_2_O_4_ nanowire were studied. Current-voltage curve characteristics can be explained by the ohmic mechanism of conductivity in the low electrical field (< 1024 V/cm). With the increase of the applied electrical field (1024 V/cm < *E* < 3025 V/cm), the Schottky emission mechanism play a dominant role. At the high electrical field (> 3025 V/cm), the current-voltage curves accord with the Poole–Frenkel conduction mechanism. A semiconductive characteristic is found in the temperature-dependent conductivity in the NiCo_2_O_4_ nanowire, and the electrical conduction mechanism at low temperature (*T* < 100 K) can be explained by Mott’s VRH Model. When the temperature is greater than 100 K, electrical transport properties were determined by the VRH and NNH hopping model. This work will be helpful to the design and performance improvement of the energy storage device based on the NiCo_2_O_4_ nanowires.

## Abbreviations

AFM: Atomic force microscope
EBL: Electron beam lithography
I-V: Current-voltage
NNH: Nearest neighbor hopping
P-F: Poole–Frenkel
PMMA: Polymethylmethacrylate
SEM: Scanning electron microscope
TEM: Transmission electron microscope
VRH: Variable range hopping
